# Cocoa Flavonoids Reduce Inflammation and Oxidative Stress in a Myocardial Ischemia-Reperfusion Experimental Model

**DOI:** 10.3390/antiox9020167

**Published:** 2020-02-18

**Authors:** Sajeela Ahmed, Naseer Ahmed, Alessio Rungatscher, Daniele Linardi, Bibi Kulsoom, Giulio Innamorati, Sultan Ayoub Meo, Mebratu Alebachew Gebrie, Romel Mani, Flavia Merigo, Flavia Guzzo, Giuseppe Faggian

**Affiliations:** 1 Department of Surgery, Division of Cardiac Surgery, University of Verona, 37129 Verona, Italydr.naseer99@gmail.com (N.A.);; 2 Department of Biological and Biomedical Sciences, Aga Khan University, 74800 Karachi, Pakistan; 3 Department of Biochemistry, Jinnah Medical & Dental College, 74800 Karachi, Pakistan; 4 Department of Physiology, College of Medicine, King Saud University, 11461 Riyadh, Saudi Arabia; 5 Department of Biomedicine, Division of Histology, University of Verona, 37134 Verona, Italy; 6 Department of Biotechnology, Division of Biology and Botany, University of Verona, 37134 Verona, Italy

**Keywords:** flavonoids, cocoa extract, ischemia-reperfusion injury, oxidative stress, apoptosis, inflammatory markers

## Abstract

Consumption of flavonoid-rich nutraceuticals has been associated with a reduction in coronary events. The present study analyzed the effects of cocoa flavonols on myocardial injury following acute coronary ischemia-reperfusion (I/R). A commercially available cocoa extract was identified by chromatographic mass spectrometry. Nineteen different phenolic compounds were identified and 250 mg of flavan-3-ols (procyanidin) were isolated in 1 g of extract. Oral administration of cocoa extract in incremental doses from 5 mg/kg up to 25 mg/kg daily for 15 days in Sprague Dawley rats (*n* = 30) produced a corresponding increase of blood serum polyphenols and become constant after 15 mg/kg. Consequently, the selected dose (15 mg/kg) of cocoa extract was administered orally daily for 15 days in a treated group (*n* = 10) and an untreated group served as control (*n* = 10). Both groups underwent surgical occlusion of the left anterior descending coronary artery and reperfusion. Cocoa extract treatment significantly reversed membrane peroxidation, nitro-oxidative stress, and decreased inflammatory markers (IL-6 and NF-kB) caused by myocardial I/R injury and enhanced activation of both p-Akt and p-Erk1/2. Daily administration of cocoa extract in rats is protective against myocardial I/R injury and attenuate nitro-oxidative stress, inflammation, and mitigates myocardial apoptosis.

## 1. Introduction

In United States, about one million people suffer from myocardial infarction per year [1]. Ischemic heart disease provoked by limited blood supply to cardiomyocytes upon occlusion of coronary vessels is the main precursor of myocardial infarction. Prevention of tissue damage in patients with ischemic heart disease can be effectively accomplished through reperfusion of the ischemic myocardial tissue [2,3]. However, reperfusion itself is responsible for inducing injury to cardiomyocytes via multiple mechanisms, leading to heart failure [4,5]. Oxidative stress and myocardial inflammatory pathways play a leading role in the pathogenesis of myocardial ischemia-reperfusion (I/R) injury [6]. Oxidative stress is caused by an intracellular redox imbalance between pro- and anti-oxidants [7,8,9]. Exogenous antioxidants might influence the course of the ischemic heart disease by providing therapeutic substances which help in restoring and maintaining a balanced system [10]. Therefore, plant resources with anti-oxidant activity would be worthy natural substances for protection against ischemic heart disease. The progression of reperfusion, thus, is accompanied by the development of oxidative stress with the generation of free radicals and leukocyte activation that lead to myocytes apoptosis [11]. Exogenous antioxidants might influence the course of the ischemic heart disease by contributing to restoring a balanced system. 

Many scientists have been working to explore antioxidant-rich natural sources to protect against myocardial I/R injury [12]. Many studies have reported plants as the main reservoir of natural antioxidants and anti-inflammatory compounds, thus pointing towards the protective role of plant products against inflammatory and reperfusion injury [12,13,14,15]. 

Cocoa and chocolate products contribute high levels of flavonoids among commonly consumed foods and have been historically used as a medicine to cure inflammation, pain, and numerous other diseases [13,16]. It is observed that adipose tissue inflammation can be reduced by long-term cocoa supplementation. In a recent study reported by Akinmoladun et al., antioxidant-containing extracts of cocoa and the kola nut tree have shown a protective effect against myocardial I/R injury using Langerdorff-perfused rat hearts [13]. In another study, flavonoids (5-hydroxy derivatives: 5-hydroxy flavone, apigenin, chrysin, and naringenin) lowered myocardial tissue injury and improved post-ischemic functional recovery [17]. Earlier, an in vitro study reported reduced IL-1 mRNA expression and IL-2 secretion by T-cells in polyphenol containing cocoa liquor [18]. However, previous studies lack biochemical analysis of inflammatory markers and signaling protein activities, and evaluation of myocardial nuclei apoptotic levels in rat hearts after I/R injury. Moreover, to the best of our knowledge, dose optimization of the cocoa extract in blood sera in rats has not been documented previously in literature. 

In this study, we investigate the effects of a commercially available cocoa extract on oxidative stress, inflammation, and apoptosis to ascertain whether it can protect myocardium in an in vivo experimental model of I/R injury.

## 2. Methods and Materials

### 2.1. Chemicals and Materials Used

CocoaVia^®^ was purchased from Mars Inc., (Hackettstown, NJ, USA). Primary antibodies Nitro-tyrosine, IL-6, NFkB2, P-Erk, P-akt antibody were obtained from Bioss antibodies, Novusbio, and Cell Signaling Technology (Danvers, MA USA). In Situ Cell Death Detection Kit, AP was from Roche (Basel, Switzerland).

### 2.2. Animals

Thirty healthy Sprague Dawley (SD) male rats of average weight 300–350g were used for the dose response experiment and 20 rats were used to assess the effect of cocoa extract on ischemia reperfusion injury. All animal experiments were done per the ethical guidelines reviewed and approved by “University of Verona Ethical Committee and the Italian Ministry of Health (341/2016-PR) at C.I.R.S.A.L. (Interdepartmental Research Centre for Laboratory Animals) of the Biological Institutes, University of Verona, and Verona, Italy”.

### 2.3. Extraction and Identification of CocoaVia^®^ Contents

Total phenolic compounds of the cocoa extract supplement (Cocoavia; Mars Inc., Hackettstown, NJ, USA) made by patented process (Cocoapro; Mars Inc., Hackettstown, NJ, USA) were extracted with three volumes of ice-cold methanol (*w/v*)). Samples were mixed, sonicated (15 min, 4 °C), centrifuged (16,000× *g* rpm, 10 min, 4 °C), and filtered through 0.2 µm pore filters. The supernatant samples were diluted 1:100 and 1:10 (*v/v*) with LC-MS grade methanol for HPLC-ESI-MS. The samples were further diluted 1:2 with LC-MS grade water and passed through Minisart 0.2 µm filters following protocol [19]. Each sample was analyzed in two technical and three biological replicates, with 20 µL injection volumes. The unknown metabolites of samples were analyzed using HPLC-ESI-MS (Esquire 6000, Bruker Daltonics, Billerica, USA), followed by ESI (Electrospray Ionization)-base peaks, MS/MS, and MS3 fragmentation at its retention time and mass to charge ratio (*m/z*). For this purpose, an “in house” library of commercial standard spectra, scientific literature, and online databases such as Mass bank (www.massbank.jp) were used. The quantification of the metabolites in the methanolic extracts was carried out through HPLC-DAD (Beckman Coulter Gold 126 Solvent Module coupled with a Gold 168 Diode Array Detector), relying on the calibration curves of authentic standard compounds.

### 2.4. Quantification of Polyphenols Levels in Blood Samples

Thirty rats were divided into six groups of five rats each. Among them, group 1 served as control, fed with normal rat diet, while the animals of other groups were administrated oral gavage with 5, 10, 15, 20, and 25 mg/kg body weight of cocoa extract dissolved in water, five times per week for 15 days, respectively. All animals were kept at a temperature of 22–24 °C and fed with a regular pellet diet ad libitum.

A total of 1 mL of blood was collected, 1 hour after oral administration of cocoa extract powder from each rat, which showed a higher concentration of flavonoids in plasma between 30 and 60 min through lateral tail vein [19]. Blood was deposited in clean heparinized glass tubes, centrifuged (3500 rpm for 15 min), and stored at −80 °C for the HPLC-ESI-MS analysis. The total amount of flavonoid in blood sera from each sample were calculated by HPLC-ESI-MS [19,20].

### 2.5. Induction of Ischemia/Reperfusion Injury and Tissue Collection

**Figure 1:**
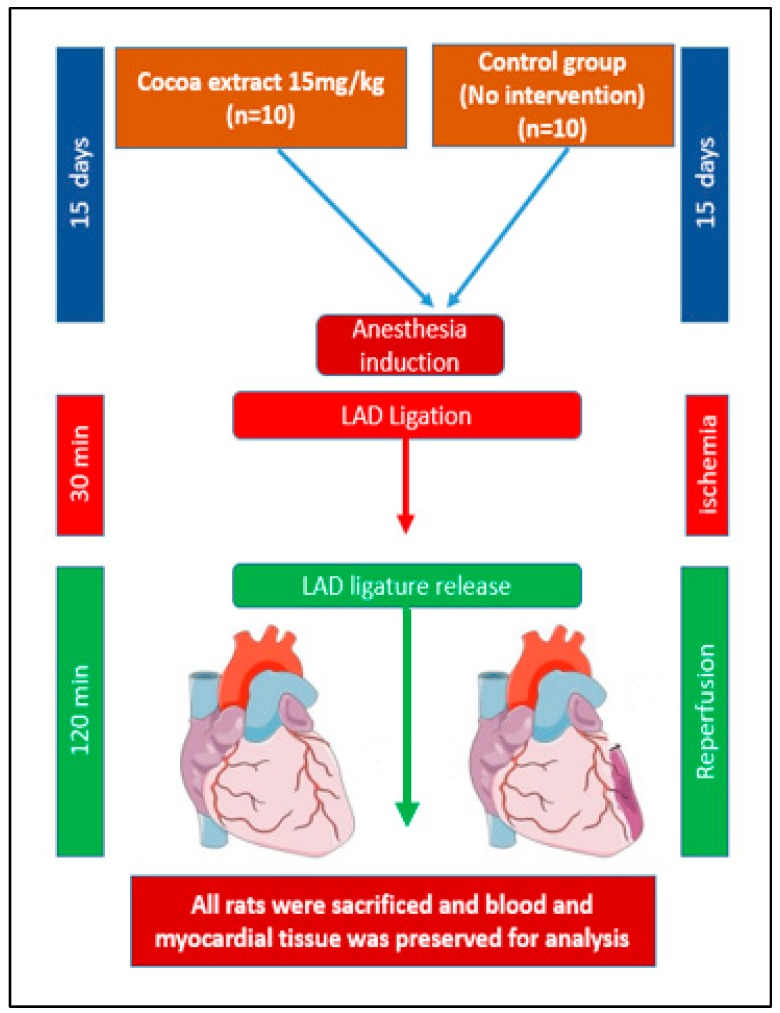
Schematic experimental design.

Twenty rats were divided into two groups, control and treated, with ten (10) in each group. The selected dose of 15 mg/kg of cocoa from our previous results was given to the treated group (*n* = 10) once a day for 15 days. The control group remained untreated. Rats (*n* = 20) were anesthetized with 5% isoflurane in 50% O_2_ administered through a facial mask and maintained anesthesia using 2% isoflurane throughout procedure. These anesthetized animals were subjected to ischemia for 30 min by left anterior descending (LAD) coronary artery ligation, followed by 120 min reperfusion, as previously described [21]. The onset of ischemia was confirmed when cyanosis developed on the wall of the ischemic myocardium, as evidenced by saddleback-type (ST) segment elevation and a significant T-wave increase recorded on the electrocardiograph Power Lab data acquisition system (model ML866, AD Instruments, Colorado Springs, CO) and Animal Bio Amp (model ML136, AD Instruments)). Then, the ligation was opened to allow reperfusion to the ischemic part of the myocardium for 120 min. Hearts were excised as demonstrated in Figure 1, the ischemic part of the left ventricle was split into two: one portion was stored in 4% formalin for immunohistochemistry and Terminal Deoxynucleotidyl Transferase dUTP Nick End Labeling (TUNEL) analysis, the second portion was stored in liquid nitrogen for of nitro-oxidative stress measurements. Experimental design is illustrated in Figure 1.

### 2.6. Immunohistochemistry Staining

To access the inflammation and nitrative stress and signaling pathways activation in myocardial tissue, samples (*n* = 10 each group) were embedded in paraffin. Fixed tissues were sectioned 3 μm, deparaffinized, and dehydrated with two grades of xylene and four grades of ethanol, following the method in [22]. After antigen retrieval and endogenous peroxidase activity procedure, all sections were incubated with primary antibodies, IL-6 (1:100), NF-κB2 (1:500), Nitrotyrosine (1:300), p-Erk (1:200), and p-Akt (1:200) (Sigma–Aldrich, United Kingdom), diluted in antibody diluent and kept at 4 °C for overnight. Following the incubation with primary antibodies, tissue sections were rinsed, and incubated with the biotinylated anti-rabbit secondary antibody (1:400), avidin–biotin complex substrate, and diaminobenzidine (Dako Corp., Carpinteria, CA, USA). Sections were rinsed two times and mounted after dehydration in three grades of ethanol and cleared in two grades of xylene. The negative control was used to confirm and check the absence of the signal or specificity of staining. Image acquisition of all the sections was done under Olympus System BX51 Universal research microscopy (Olympus corporation, Tokyo, Japan). The images were analyzed by using ImageJ software (NIH, Bethesda, MD, USA) to quantify the strength of immune-peroxidase staining in heart tissue. 

### 2.7. Oxidative Stress Measurement

The analysis of malondialdehyde (MDA) levels in myocardial tissue samples collected from the affected area was performed as described by Ben Maunsour et al. [23].

The reactive oxygen species (ROS) in myocardial tissue was measured using a spectrophotometry and the absorbance determined (peak at 505 nm max) value is expressed as Carr. Units, as explained by Rizzo et al. [24].

### 2.8. Terminal Deoxynucleotidyl Transferase dUTP Nick End Labeling (TUNEL) Assay

TUNEL assay is a method that labels 3′-hydroxyl termini in the double-stranded DNA fragments generated as a result of apoptosis. Paraffin-mounted heart sections from control and treated groups (*n* = 10) were deparaffinized with xylene and descending concentrations of ethanol. Apoptotic cells were stained following the protocol provided by the In Situ Cell Death Detection Kit, AP (ref. 11684809910, Version 11, Roche Diagnostics, Indianapolis, IN, USA). Hoechst 33342 solution was used for staining nuclei and examined under confocal microscope. The images were analyzed by ImageJ software (NIH, Bethesda, MD, USA), average mean fluorescent intensities (MFIs) were measured by multiplying the intensities of each image [25].

### 2.9. Statistical Analysis

Analyses were performed using SPSS software version 21 (SPSS Inc., Chicago, IL, USA). Comparison between control and experimental groups was statistically evaluated by the Students t-test, and *p*-value < 0.05 was considered statistically significant. All the results were expressed as mean ± SD.

## 3. Results

### 3.1. Identification of Phenolic Compounds

**Figure 2:**
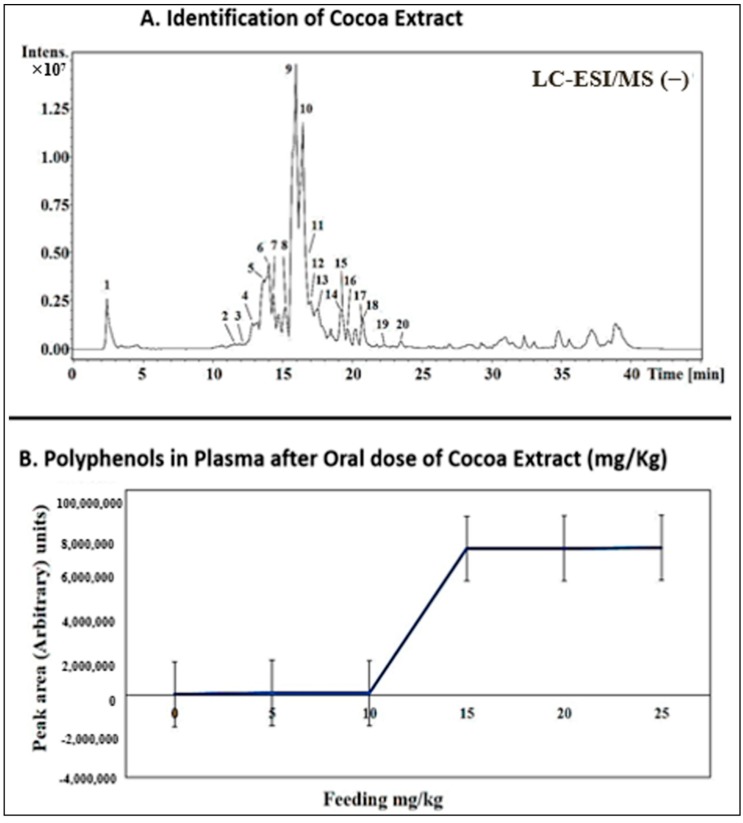
Cocoa Extract: (**A**) shows HPLC-ESI-MS graph with various peaks correlating with 19 different phenolic compounds found in cocoa extract. (**B**) shows a curve correlating different oral doses (mg/kg) of cocoa extract and blood levels of phenolic compounds in rats. It shows an optimal dose of 15 mg/kg to be the optimal dose of cocoa extract (six groups (*n* = 30) and each group (*n* = 5)).

A total of 250 mg of flavan-3-ols (procyanidin) was identified from 1 g of cocoa extract. From this, 19 different phenolic compounds were determined (Table 1 and Figure 2a).

### 3.2. Dose Response and Dose Adjustment

Oral administration of cocoa extract in incremental doses from 5 mg/kg up to 25 mg/kg daily for 15 days produced a corresponding increase in blood serum polyphenols and became constant after 15 mg/kg (Figure 2B). 

### 3.3. Anti-Inflammatory Effect of Cocoa

**Figure 3:**
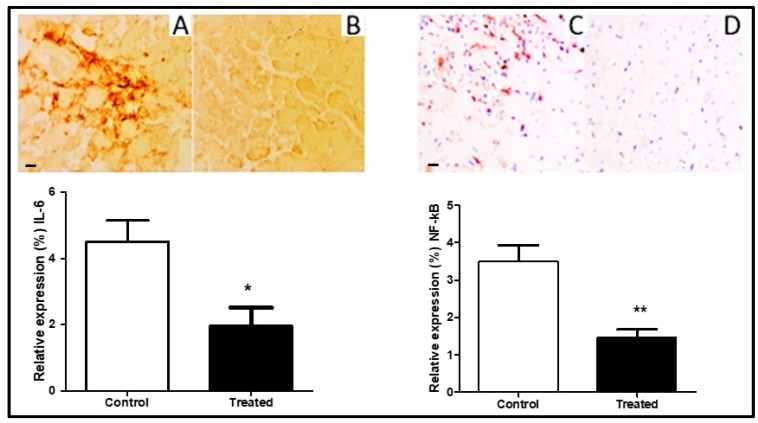
Immunohistochemical analysis showing expression (representative images, 20× magnification) of IL-6 and NF-kB2 in myocardial tissue of cocoa treated rats (*n* = 10) compared to control rats (*n* = 10), (*p = 0.0032, **p = 0.0001 respectively). presented as mean ± SD and scale bar is 100 µm. (**A**,**B**): IL-6, (**C**,**D**): NF-kB.

The extent of inflammation after I/R was measured by the expression of IL-6 and NF-kB in the myocardium. Immunohistochemistry revealed a reduced expression of IL-6 (Figure 3A,B) was significantly lowered after cocoa extract treatment 15 mg/kg (*p* < 0.01) in the ischemia-reperfusion injury model. The levels of NF-kB were remarkably low in the treated group as compared to control (Figure 3C,D).

### 3.4. Nitro-Oxidative Stress Attenuation with Cocoa

**Figure 4:**
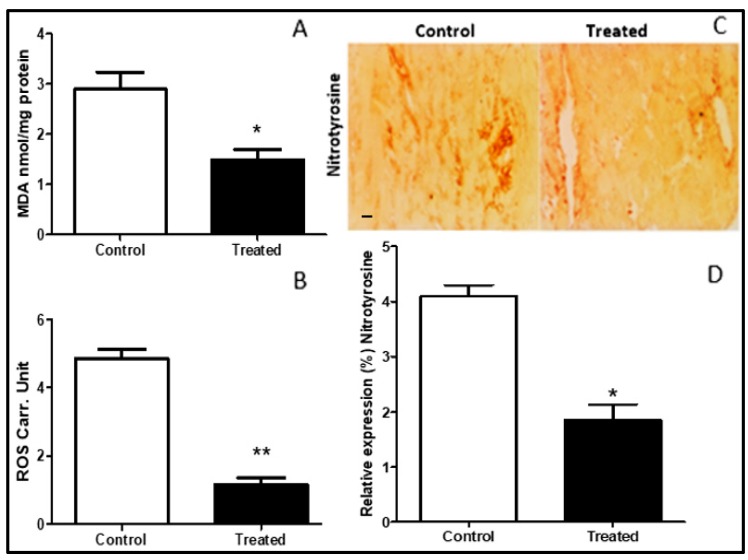
Effects of cocoa on oxidative stress on myocardial tissue rats: Lower oxidative stress was recorded in cocoa-treated rats (*n* = 10) myocardial tissue as compared to control (*n* = 10). (**A**) Lipid peroxidation index (TBARS) expressed as malondialdehyde (MDA) concentration measured in nmol/µg (*p* = 0.031), * *p* < 0.05. (**B**) Reactive oxygen species (ROS) measured as Carr. Unit (Carratelli Unit) (*p* = 0.0001), ** *p* < 0.001. (**C**,**D**) Expression of nitrotyrosine staining in myocardial tissue in the control and treated groups (20×) (*p* = 0.04), * *p* < 0.05. Data illustrated in the graphs are presented as mean ± SD and scale bar is 100 µm.

Oxidative stress was studied by identifying the levels of the “lipid peroxidation index, Thiobarbituric acid reactive substances (TBARS)”. TBARS expressed as malondialdehyde (MDA) levels was lower in the treated group as compared to control (Figure 4A). The concentration of ROS was also attenuated in the treated group as compared to control (Figure 4B). Nitrosative stress was assessed by measuring nitrotyrosine levels in myocardial tissue. The nitrotyrosine was reduced by cocoa treatment (*p* < 0.05) (Figure 4C,D). 

### 3.5. Akt and Erk1/2 Signaling Pathways Activation

**Figure 5:**
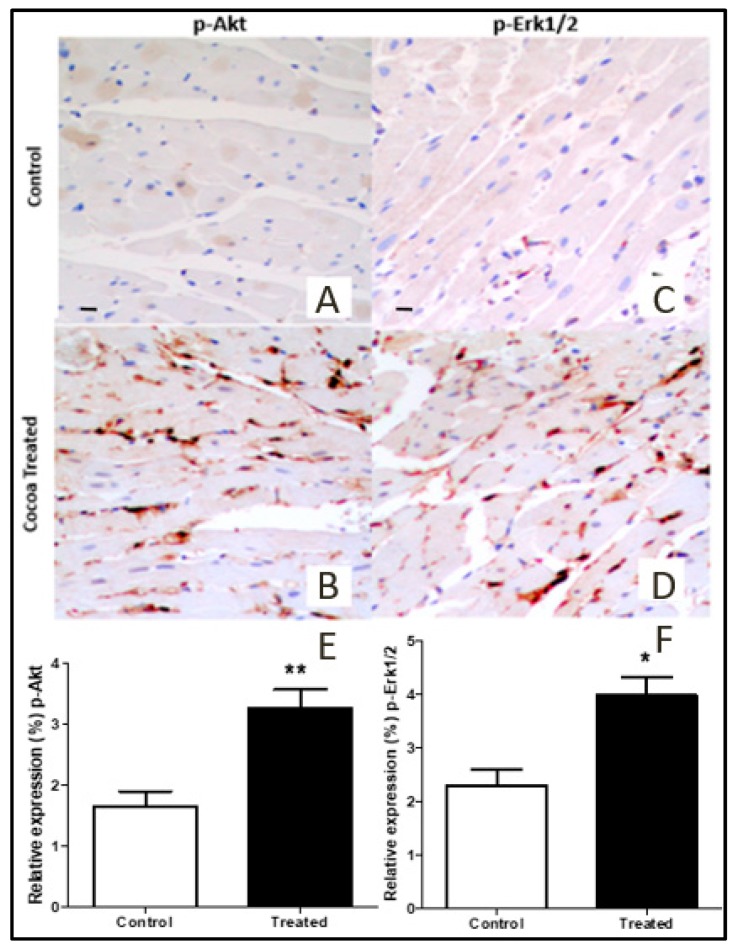
Immunohistochemical labeling of p-Akt (**A**,**B**,**E**) and p-Erk1/2 (**C**,**D**,**F**) in cocoa-treated rat myocardial tissue compared to control. The graphs show a significant elevation in the activation of p-Akt and p-Erk1/2 in the myocardial tissue of cocoa-treated rats (*n* = 10) compared to control (*n* = 10). Representative images (20×). Data illustrated in the bar graph are presented as mean ± SD. *p*-value less than 0.05 considered statistically significant. P-ERK1/2, phosphorylated extracellular signal regulated kinases ½; p-Akt, phosphorylated serine-threonine protein kinase, (* *p* < 0.05, ** *p* < 0.001).

Immuno-peroxidase analysis of p-Akt and p-ERK1/2 in myocardial tissue from rats treated with cocoa extract and control group was performed. Enhanced phosphorylation of Akt (Figure 5A,B,E) and ERK1/2 (Figure 5C,D,F) was observed as compared to control group (*p* < 0.05 in both cases).

### 3.6. TUNEL Assay

**Figure 6:**
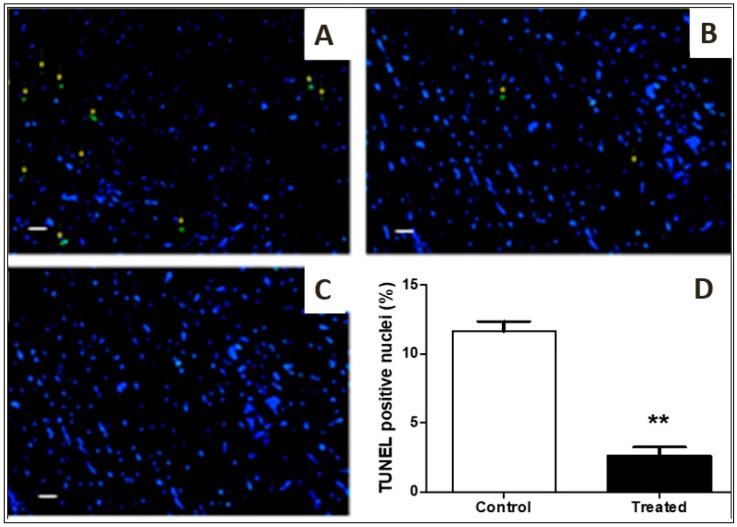
TUNEL Assay: (**A**,**B**,**C**): Representative images (*n* = 10 for each group) immunofluorescent staining for TUNEL-positive nuclei in control, treated, and negative control groups (20× magnification). TUNEL-positive myocytes were less in number in the treated group (**A**) as compared to control group (**B**). Yellow arrows indicate apoptotic nuclei and (**C**) negative control. *p*-value = 0.002 considered significant (** *p* < 0.001, control versus treated), scale bar is 100 µm. (**D**): Graphical presentation of apoptotic nuclei in control vs treated groups.

TUNEL-positive nuclei were less in number in the treated group (A) as compared to the control group (B). Yellow arrows indicate apoptotic nuclei (Figure 6A–C). There was a significant reduction in apoptosis in treated samples as compared to control (*p* < 0.001).

## 4. Discussion

Cocoa has the highest flavonol contents of all foodstuffs and its extract contains a considerable concentration of proanthocyanidins [16]. Flavonoids characterize a main division of phenolic compounds, and they are greatly active scavengers of most oxidizing molecules and free radicals involved in several diseases [17,18], including cardiovascular diseases. 

In the present study, a total of 250 mg of procyanidins was determined in 1 gram of cocoa extract. From this, 19 different phenolic compounds were identified (Figure 1 and Table 1). Many studies have shown the protective effect of cocoa in myocardial ischemia-reperfusion injury and in improving post-ischemic functional recovery [13,17]. Thus, it was observed that cocoa extract has anti-inflammatory properties [26] and it can protect against myocardial injury [27]. Even though flavonoids have many health-related benefits, their bioavailability is a major concern [28]. We hypothesized that these phenolic compounds, introduced daily with cocoa extract supplement, might contribute to reducing inflammatory markers, oxidative stress, myocardial apoptosis, and activating pro-survival pathways in the heart exposed to I/R injury. In the present study, different concentrations of a commercially available cocoa extract were investigated in a dose-response assessment in order to establish the optimal dose in a daily administration regimen in order to have maximal plasma concentration of polyphenols. An optimal dose of 15 mg/kg/BW was administered daily to investigate the possible cardioprotective effects during acute myocardial I/R.

The expression of inflammatory mediators, which recruits leukocytes into inflammatory sites, was reduced in the cocoa-treated rat heart (Figure 3). During the inflammatory process at tissue, the usual trend is the elevation of IL-6 and NF-kB levels [29]. These mediators were significantly reduced in cocoa extract-treated rat hearts. In vitro reduction in other inflammatory meditators, such as IL-1 mRNA expression and IL-2 secretion by T-lymphocyte treated with cocoa liquor was also reported [17]. Thus, reduction in inflammatory markers in cocoa-treated rat hearts suggests an anti-inflammatory and cardioprotective role of cocoa extract flavonoids.

Malondialdehyde (MDA), a marker of oxidative stress, was attenuated in cocoa-treated heart tissue (Figure 4A). The levels of reactive oxygen species were found to be significantly higher in cocoa-treated rat myocardium as compared to the control group after the induction of I/R (Figure 4B). Similarly, Akinmoladun et al. explored the protective effects of cocoa and kola nut tree extracts, and also reported a reduction in oxidative stress and post I/R injury in an in-vitro model [13]. Nitric oxide (NO) produced by endothelial nitric oxide synthase (eNOS) constitutively is reduced in ischemia but inducible NOS is activated by I/R, thus increasing nitrosative stress. Nitrotyrosine (peroxynitrite) levels, which indicate nitrosative stress [30] in myocardial tissue due to I/R, were lower in cocoa-treated rat heart in our study (Figure 4C,D).

The ultimate step of myocardial injury in reperfusion injury is apoptosis, and inhibition of apoptotic pathways demonstrated significant cardioprotection. Reduced apoptosis leads to reduced myocardial injury [31]. Apoptosis could be due to mitochondrial dysfunction, which results in low energy for myocyte contraction. Additionally, there may be an increase in oxidative stress that directly damages and induces apoptosis in myocytes [32,33,34]. During I/R, the fuel preference switches to glucose. This alteration safeguards the heart, in part, because “free acids waste more oxygen to be oxidized”. However, over time, while not oxidized, fatty acids hoard inside the myocytes, leading to cardiac lipotoxicity [35,36]. Nitrite reacts with critical thiols to form nitrosothiols, which act as antioxidants that prevent the irreversible oxidation of proteins and lipids during the early oxidative burst of reperfusion” [37,38].

Despite the pharmacological effectiveness of cardioprotective drugs, novel products which inhibit ischemic organ damage are required and care is being given to discovering novel pharmacological agents from plants [39]. Infact, the medicinal plants’ antioxidant content may contribute to protection from diseases. Commonly found in plants, phenolic compounds are major antioxidant phytochemicals [27]. When the plants are consumed, these phytochemicals contribute to the intake of natural antioxidants in the diets of animals as well as humans.

The reduced levels of myocardial apoptosis observed in cocoa-treated rats demonstrate potential effect of flavonoids containing cocoa in reducing myocardial apoptosis. The toxicological study also indicated the contribution of epicatechin and catechin in cocoa in the reduction of apoptosis via the inhibition of amyloid-β protein [40]. The reduced myocardial apoptosis could be due to the antioxidant content of cocoa, which might contribute to the protection against oxidative stress produced by I/R.

In our study, immuno-peroxidase analysis of myocardial tissue from rats treated with cocoa extract showed elevation in the activity of p-Erk1/2 and p-Akt (Figure 5). Pro-survival pathways proteins (Erk1/2 and Akt) are known for their contribution to cell survival during ischemia-reperfusion injury. Enhanced phosphorylation of Erk1/2 and Akt were observed with cocoa treatment, which leads to cardioprotection [41]. The TUNEL assay, which is good indicator to measure apoptosis, demonstrated reduced apoptosis in cocoa-treated heart tissues as compared to control (Figure 6).

Therefore, this study suggests that treating rats with cocoa extract significantly attenuates inflammation, oxidative stress, and apoptosis in the myocardium and the process can be modulated by the activation of Erk1/2 and Akt pathways.

The present study investigated only one commercially available cocoa extract. Other products could give different results. Additionally, we investigated healthy rats; in order to translate the results to humans they should be confirmed in a randomized clinical study in patients with ischemic cardiomyopathy taking different medications.

## 5. Conclusions

Daily supplementation of cocoa extract attenuates myocardial I/R injury, limiting oxidative and nitrosidative stress and inflammation with a reduction in myocardial apoptosis.

## Figures and Tables

**Table 1:** Phenolic molecules identified from the cocoa extract samples analyzed by chromatographic mass spectra in negative ion mode.

No.	(−) *m/z*	No.	(−) *m/z*
1	Di-hexose, Di-hexose (2M−H)	11	Procyanidin pentamer
2	*N*-[3′,4′-dihydroxy-(*Z*)-cinnamoyl]-l-aspartic acid	12	Procyanidin monomer
3	Catechin hexose	13	Procyanidin aptamer
4	Catechin	14	Procyanidin hexamer
5	Procyanidin trimer	15	Procyanidin
6	Procyanidin tetramer	16	Quercetin hexose
7	Procyanidin B2	17	Procyanidin derivative
8	Epicatechin	18	Ellagic acid pentose
9	Procyanidin trimer, Catechin derivative	19&20	(Epi) catechin ethyl trimmers
10	Procyanidin tetramer		
